# Supplementary material for: Polyketide synthase gene clusters in the ericoid mycorrhizal fungus Oidiodendron maius: genome-wide prediction, expression profiling, and functional characterization of knockout mutants
Source: Front Plant Sci. 2026 Sep 16;17:1924430. doi: 10.3389/fpls.2026.1924430 (PMC13623903; doi:10.3389/fpls.2026.1924430)
Supplement: Supplementary file 1 [file SupplementaryFile1.zip › Supplementary Materials/Supplementary Folder 1.zip!/Oidma1_AssemblyScaffolds_Repeatmasked/knownclusterblast/cluster42/jgi.p_Oidma1_59451_mibig_hits.html]

| MIBiG Protein | Description | MIBiG Cluster | MiBiG Product | % ID | % Coverage | BLAST Score | E-value |
| --- | --- | --- | --- | --- | --- | --- | --- |
| BAE56602.1 |  | BGC0001123 | Aspirochlorine\_biosynthetic\_gene\_cluster | 54 | 56.8119891008 | 463 | 5.8e-130 |
| ctg1\_orf0002 |  | BGC0000321 | Chaetocin\_biosynthetic\_gene\_cluster | 44 | 55.0408719346 | 362 | 1.4e-99 |
| BAE56609.1 |  | BGC0001123 | Aspirochlorine\_biosynthetic\_gene\_cluster | 43 | 56.1307901907 | 356 | 1e-97 |
| EAU36746.1 | conserved\_hypothetical\_protein | BGC0000292 | Acetylaranotin\_biosynthetic\_gene\_cluster | 53 | 39.6457765668 | 314 | 4.4e-85 |
| AAS92548.1 | SirM | BGC0001044 | Sirodesmin\_biosynthetic\_gene\_cluster | 37 | 56.6757493188 | 295 | 2.1e-79 |
| AAD32742.2 | MmcR | BGC0000915 | Mitomycin\_biosynthetic\_gene\_cluster | 26 | 48.6376021798 | 109 | 1.6e-23 |
| CAM34360.1 | putative\_O-methyltransferase | BGC0000242 | Lysolipin\_biosynthetic\_gene\_cluster | 30 | 42.7792915531 | 106 | 1.8e-22 |
| AME18001.1 | O-methyltransferase | BGC0001378 | Tiancimycin\_biosynthetic\_gene\_cluster | 28 | 44.1416893733 | 103 | 1.2e-21 |
| ADE22330.1 | O-methyltransferase | BGC0000065 | Galbonolides\_biosynthetic\_gene\_cluster | 26 | 46.3215258856 | 100 | 7.6e-21 |
| AEE65480.1 | putative\_O-methyltransferase | BGC0000223 | Fluostatin\_biosynthetic\_gene\_cluster | 25 | 47.8201634877 | 97 | 8.4e-20 |
| AKD43502.1 | O-Methyltransferase | BGC0001409 | Dutomycin\_biosynthetic\_gene\_cluster | 35 | 21.2534059946 | 94 | 5.5e-19 |
| BAB69170.1 | O-methyltransferase | BGC0000271 | Spore\_pigment\_biosynthetic\_gene\_cluster | 25 | 47.5476839237 | 86 | 1.9e-16 |
| AFY23046.1 | TamO | BGC0000274 | Tetarimycin\_biosynthetic\_gene\_cluster | 26 | 44.4141689373 | 85 | 2.5e-16 |
| AEI98654.1 | CtcK | BGC0000209 | Chlortetracycline\_biosynthetic\_gene\_cluster | 24 | 48.3651226158 | 85 | 2.5e-16 |
| AAM54085.1 | methyltransferase | BGC0000020 | Ansamitocin\_biosynthetic\_gene\_cluster | 25 | 44.2779291553 | 80 | 8.2e-15 |
| AAP69580.1 | putative\_O-methyltransferase | BGC0000226 | Gilvocarcin\_biosynthetic\_gene\_cluster | 26 | 21.1171662125 | 65 | 2.7e-10 |
| ABX71108.1 | Lct25 | BGC0000238 | Lactonamycin\_biosynthetic\_gene\_cluster | 28 | 22.4795640327 | 61 | 6.7e-09 |
